# The Implication of Misinformation and Stigma in Age-Related Quality of Life, Depression, and Coping Mechanisms of Adult Patients with Psoriasis

**DOI:** 10.3390/medicina58101420

**Published:** 2022-10-09

**Authors:** Luminita Decean, Mihai Badea, Victoria Rus, Gabriela Buicu, Andreea Sasu, Ciprian Nicolae Pilut, Adriana Mihai

**Affiliations:** 1 Department of Microbiology, George Emil Palade University of Medicine, Pharmacy, Science and Technology, Strada Gheorghe Marinescu 38, 540139 Targu Mures, Romania; 2 Multidisciplinary Research Center on Antimicrobial Resistance (MULTI-REZ), Microbiology Department, “Victor Babes” University of Medicine and Pharmacy, Eftimie Murgu Square 2, 300041 Timisoara, Romania

**Keywords:** psoriasis, quality of life, depression, stigma, misinformation, COPE, DLQI, SF-12

## Abstract

*Background and Objectives*: Stigma and lack of acceptance in society might have detrimental effects on the quality of life of patients with psoriasis, sometimes being comparable with other chronic diseases and conditions that affect the appearance of a patient, such as burns. Therefore, we surveyed our patients diagnosed with psoriasis to determine the implications of misinformation and stigma for their quality of life, depression, and coping strategies stratified by different age categories. *Materials and Methods*: A cross-sectional study was designed for a sample size of a minimum of 45 patients considering a prevalence of psoriasis of 2–3% in the general population. The study participants (patients and controls) were given both a paper-based unstandardized questionnaire and an online version of three standardized surveys. The cohort of patients was further split into three age groups to determine their age-related quality of life and coping mechanisms. *Results*: The proportion of patients with a history of depression and depressive symptoms among patients with psoriasis was significantly higher. Multiple discrepancies were observed between patients and controls regarding questions that targeted stigma and misinformation. On the Coping Orientation to Problems Experienced Inventory (COPE-60) questionnaire, older patients were more likely to use positive coping mechanisms such as engagement and problem-focused coping, while the young patients were using more emotion-focused coping mechanisms. However, patients in the 30–50 age range group scored the highest on physical and mental health among all participants who filled the 12-Item Short Form Survey (SF-12) survey. The Dermatology Life Quality Index (DLQI) results showed significantly more patients answering “a lot and very much” concerning embarrassment and social activities, while sexual difficulties affected the older patients. The strongest correlations with depression were observed in the young patient group, who believed that psoriasis can cause skin cancer (rho = 0.418) and who had sexual difficulties (rho = 0.414) and embarrassment (rho = 0.359) as evaluated by the DLQI survey. In the 30- to 50-year-old group, the strongest correlations were with the feeling of being stigmatized (rho = 0.376), having sexual difficulties (rho = 0.367) and disengagement coping style (rho = 273). *Conclusions*: While the respondents are reasonably well-informed regarding psoriasis, a degree of stigma remains, likely due to involuntary emotional responses such as repulsion and embarrassment. It is essential to establish initiatives aimed at educating the general public, raising awareness, and establishing a more tolerant social environment for psoriasis patients.

## 1. Introduction

Psoriasis is one of the most widespread immune-mediated diseases that affects the skin and joints [1]. The prevalence of the disease worldwide is approximately 2%, but it varies depending on the region [2]. It is a chronic inflammatory disease accompanied by systemic inflammation that leads to the development of comorbidities, especially cardiovascular diseases, obesity, and metabolic syndrome but also mental disorders such as anxiety and depression, with a huge impact on the quality of life of patients [3].

Experiences of humiliation and rejection and negative impacts on employment capacity, which have consequences on the quality of life of these patients, have been described in the literature. Stigmatization can exacerbate negative emotions and unfavorable self-perceptions such as low self-esteem and a negative body image [4,5]. Stigmatization not only leads to the social exclusion of patients with psoriasis but also affects their mental status, possibly causing anxiety, anger, depression or its most serious complication, suicide [6,7,8,9].

According to the stress–coping model, a patient’s own underlying views of disease, body shame, and unpleasant experiences of social contact may have a particularly important role in the patient’s ability to cope with the condition. Divergent assessments and beliefs about the impact of psoriasis on patients’ day-to-day life can have an effect on the doctor–patient relationship, as well as on health care, by causing patients to be unwilling to use the health care system or to distrust the therapies that have been prescribed [10,11].

In addition, it is possible that social support is of utmost significance for this specific set of patients. Psoriasis, along with other skin illnesses, continues to elicit feelings of disgust and intolerance [12]. Social support might be a significant influence in mitigating the unfavorable effects of the illnesses such as depression or depressive symptoms. In brief, the significance of the role that patients’ subjective assessments of the severity of the condition played on the quality of life will emphasized in this research, as well as the determinants for depression by age group.

## 2. Materials and Methods

### 2.1. Design and Inclusion Criteria

A multicentric cross-sectional study was conceptualized at the University of Medicine and Pharmacy Iuliu Hatieganu in Cluj Napoca, Romania. The research population and relevant features were identified using a population-based administrative database of patients who presented in the outpatient setting in the cities of Targu-Mures, Cluj-Napoca and Brasov. Patients with psoriasis were selected if they had a previous diagnosis confirmed by skin biopsy. The patients were informed, by word and in the introduction of the questionnaire, that the data being collected were anonymous and to be used for scientific purposes. The respondents’ choice to participate in the study was not constrained, and their participation did not impact the quality of medical care received at the clinic. All respondents were over 18 years of age and within their ability to offer informed consent for filling out the questionnaire, and all questionnaires were filled before the initiation of psoriasis treatment to avoid biasing the quality of life results. The research complied with the ethics criteria from the university where the study was developed and was approved by the ethics committee on 9 June 2016 with the number 252.

### 2.2. Questionnaires and Variables

Using convenience sampling, it was determined that a total of 45 psoriasis cases were adequate for representing the general population considering a prevalence of 2–3% of psoriasis in the Romanian adult population [13], a confidence level of 95% and a margin of error of 5%. Those who agreed to participate were split into patients and controls, while the cohort of patients was further split into three age groups to determine their age-related quality of life and coping mechanisms. The paper-based unstandardized questionnaire was printed on size A4 paper with colored illustrations and then distributed in three clinics in Targu-Mures, Cluj-Napoca and Brasov to be offered to patients awaiting their consult. Patients who agreed to complete the short paper questionnaire were asked to consent to participation in the online part of the study that comprised three standardized surveys translated and adapted for Romanian: (1) Coping Orientation to Problems Experienced Inventory (COPE-16) [14,15]; (2) 12-Item Short Form Survey (SF-12) [16,17]; and (3) the Feelings of Stigmatization Questionnaire, Dermatology Life Quality Index (DLQI) language [18].

The variables considered for statistical analysis comprised the background data from patients and controls (age group, gender, area of residence, relationship status, level of income and level of education (defined against the national average), occupation, substance use behavior, history of major depression or current depressed mood or anhedonia), questions related to misinformation and stigmatization, COPE-60 survey, SF-12 survey and the DLQI questionnaire.

### 2.3. Statistics

The collected information was input in a Microsoft Excel database. Descriptive statistics results were computed in Microsoft Excel and are expressed in percentages of the entire population included in the study. The inferential statistical analysis was performed with the IBM SPSS software, v27.0. (SPSS. Inc., Chicago, IL, USA). Categorical data were analyzed by applying the Chi-square test and Fisher’s test. Normally distributed data are presented as means and standard deviation, being analyzed with ANOVA. Correlation coefficients were calculated using Spearman’s rank correlation analysis. The statistical significance threshold chosen was alpha = 0.05, and the p-value was considered statistically significant when *p* < alpha. The results were expressed in the form of absolute frequencies and percentages.

## 3. Results

### 3.1. Background Analysis

At the end of the study, we collected a total of 218 complete surveys from patients with psoriasis (cases) and 374 from patients without psoriasis (controls) who presented for appointments in the outpatient setting. Table 1 presents the comparison of background data from patients and controls. There were 103 patients with psoriasis in the 18–30 age group, 98 patients in the 30–50 age group and 77 patients older than 50, with no significant differences in proportions compared with the control group of patients without psoriasis. It was observed that the majority of patients were men (56.7%), and most resided in urban areas (57.0%). The level of income and level of education in the study cohort did not have significant differences between groups, although there were more married patients and more employed patients in the control group (69.8% vs. 61.5%, *p*-value = 0.038), respectively (73.8% vs. 66.1%, *p*-value = 0.045). Lastly, the background analysis of patients identified a statistically significant difference in proportion of patients with a history of depression and depressive symptoms among patients with psoriasis (19.3% vs. 13.7%, *p*-value = 0.044).

### 3.2. Unstandardized Questionnaire

The analysis of the responses to the unstandardized questionnaire filled out by patients and controls, presented in Table 2, identified many significant differences in their answers that highlighted misinformation and stigmatization. It was observed that 28.1% of controls thought it is not safe to shake hands with someone with psoriasis, compared with only 6.0% among patients (*p*-value < 0.001). Moreover, 31.6% of controls believed that psoriasis can be caused by a lack of hygiene, and 55.1% of them would not hire someone with psoriasis for hand working jobs. A total of 7.3% of patients with psoriasis believed that it can be caused by sun exposure, compared with 13.4% of controls (*p*-value = 0.024), while also believing that it can be sexually transmitted (5.0% vs. 17.1%, *p*-value < 0.001). A high number of respondents in the control group confirmed feeling repulsion when they saw psoriatic lesions (15.8% vs. 9.2%, *p*-value = 0.001). Lastly, 37.2% of all patients with psoriasis said they feel stigmatized, compared with 27.3% similar answers from the control group (*p*-value = 0.012).

### 3.3. Standardized Surveys

**Figure 1:**
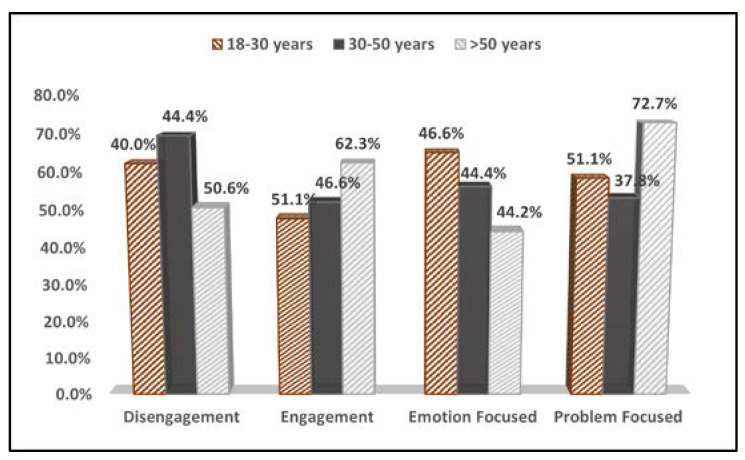
Comparison of COPE-60 questionnaire results by age group.

The comparison of COPE-60 questionnaire results stratified by the age of participants identified that patients in the 30–50 age group were more likely to use negative coping mechanisms such as disengagement (69.4%), although they were less emotion-focused (56.1%). Older patients were more likely to use positive coping mechanisms such as engagement and problem-focused coping, as seen in Table 3. The young patients used more emotion-focused coping mechanisms when dealing with their disease (65.0% vs. 56.1% vs. 44.2%, *p*-value = 0.020), as presented in Figure 1.

**Figure 2:**
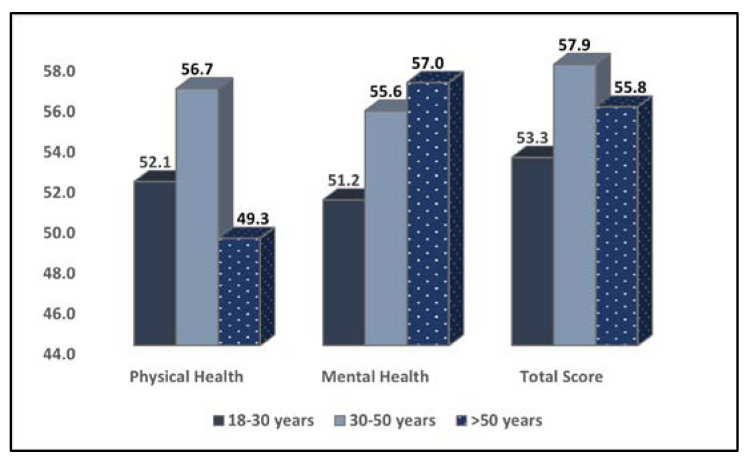
Comparison of quality of life physical and mental scores based on the SF-12 questionnaire stratified by age group.

The comparison of quality of life physical and mental scores based on the SF-12 questionnaire stratified by age group is presented in Table 4. It was observed that patients in the 30–50 age range group scored the highest on physical and mental health among all participants. As seen in Figure 2, the average total score was 57.9 in the 30–50 age group, compared with 53.3 in the young group and 55.8 in those older than 50 years (*p*-value < 0.001). The older patients, however, had the lowest level of life quality assessed by physical health test (49.3, compared with 52.1 in the young group and 56.7 in the 30–50 group (*p*-value < 0.001).

The results of the Dermatology Life Quality Index (DLQI) presented in Table 5 identified three items that were significantly different between the three age groups. Item 2 evaluated embarrassment and found that it was the highest in young adults between 18 and 30 years old (35.9%), compared with the 30–50 age group (21.4%) and those older than 50 (23.4%, *p*-value = 0.046). Item 5 compared the social activities and revealed a statistically significant difference between the patient groups answering “a lot and very much”, as presented in Table 5. Lastly, Item 9 evaluated the sexual difficulties, and here, older patients answered significantly more often with “a lot and very much” (28.6%), compared with the 30–50 age group (18.4%), and the 18–30 patients (11.7%, *p*-value = 0.015).

### 3.4. Correlation Analysis

Spearman’s rank correlation coefficients by age groups, presented in Table 6, were calculated for the dependent variable that evaluated the presence of depression and depressive symptoms in patients with psoriasis. The strongest associations were observed in the young patient group, who believed that psoriasis can cause skin cancer (rho = 0.418) and who had sexual difficulties (rho = 0.414) and embarrassment (rho = 0.359) as evaluated by the DLQI survey; all showed positive and statistically significant correlations with the dependent variable. In the 30- to 50-year group, the strongest correlations were the feeling of being stigmatized (rho = 0.376), having sexual difficulties (rho = 0.367), and disengagement coping style (rho = 273). Lastly, the strongest correlations identified in patients older than 50 years were a low physical health score on the SF-12 (rho = 0.356), having sexual difficulties (rho = 0.314), and believing psoriasis can cause skin cancer (rho = 0.311).

## 4. Discussion

### 4.1. Current Findings and Literature Analysis

The current study identified that young adults between 18 and 30 years old are the most impacted age category of patients with psoriasis due to stigmatization and that it is likely to determine the development of depression. On the opposite side, patients older than 50 years are less likely to develop stigma-related depression but are more prone to be misinformed. The impact that psoriasis has on a person’s quality of life has been a significant focus of investigation. In the current research, we were able to establish that demographic characteristics as well as psychometric factors impacted the psychosocial status of people who have psoriasis and that stigmatization is a significant issue that is present in many patients who have psoriasis. The quality of life in this particular patient population seemed to peak in the 30–50 age group, where coping mechanisms were more positive. Moreover, the data derived from our study paint an optimistic picture for patients with psoriasis since the majority of respondents were in agreement that psoriasis is not contagious or a result of poor hygiene habits, and most of them were likely to maintain friendly relations with people suffering from psoriasis.

However, on closer analysis, the descriptive data suggest that while the respondents did not wish to appear as discriminating against people with psoriasis, a degree of social exclusion was likely in real life. This is evident with regards to the questions pertaining to social interactions, since 83.2% of respondents reported that they would probably maintain a friendship with a person with visible psoriasis lesions, but only 71.9% would shake hands with their friend suffering from psoriasis. Moreover, while 82% of respondents were aware that psoriasis lesions are not contagious, only 55.1% would probably hire a person with psoriasis lesions for services such as manicure or massage. Regarding close social interaction and sexual activity, many believed this disease can be passed this way.

Inferential analysis of our data suggests that women living in urban areas are more likely to be well-informed and accepting towards people with visible psoriasis lesions. On the other side, respondents in rural areas were more likely to be misinformed, and as such, discriminate against patients with visible psoriasis lesions. Respondents identifying as male were less informed than women regarding psoriasis, with discriminatory attitudes of men being oriented towards platonic social interactions such as friendship or employment. However, another research performed with more than 100 individuals with psoriatic disease found that there was no correlation between the amount of stigmatization and either clinical or demographic characteristics [19]. The only exception was significantly greater levels of stigmatization experienced by patients who did not come from a family with a history of psoriasis. Additionally, significant correlations were identified between quality of life and most aspects of stigmatization using the 33-item Feelings of Stigmatization Questionnaire and the 6-item Stigmatization Scale to measure stigmatization.

The higher rate of acceptance of psoriasis lesions in our study by the female respondents may be explained by their prior struggles with insecurity and body-related stigmatization. A study including 188 female respondents suffering from psoriasis or obesity or as part of a control group reveals that hostile behavior is experienced more often by female patients suffering from psoriasis compared with controls. However, the respondents in the obesity group were faced with unfriendly behavior, confusion, staring and hostility [20].

While medical practitioners seek to understand the struggles of patients diagnosed with certain conditions through well-documented scientific studies, social media platforms are rapidly becoming a source of information and validation—or misinformation and stigma—for the general population. A review carried out in 2021 took this into consideration and included platforms such as TikTok, Twitter, Instagram and Facebook in their search for baseline data. Their efforts revealed that there is misinformation regarding the etiology of psoriasis, as well as promotion of untested natural “cures” for psoriasis. These false claims circulating on popular social media channels may be detrimental to the social experience of patients suffering from psoriasis [21].

A meta-analysis of 18 studies of 200,000 patients revealed that 70% of them described a stressful event occurring 12 months before the initial manifestations of their disease. This further supports the link between mental health and psoriasis. With the third tier of Maslow’s hierarchy of needs being represented by affection and friendship, it stands to reason that difficult social interactions fueled by misinformation and marked by hostility will affect patients’ mental health. Indeed, patients describe a downward spiral: as the psoriasis plaques extend and become more noticeable, patients experience discrimination more readily, up-regulating the level of stress and increasing the chance for more noticeable manifestations of psoriasis. The solution for this downward spiral lies in identifying the particular ways in which patients are ostracized and designing interventions in order to dispel misinformation and desensitize the general population to the appearance of psoriasis plaques. Another study performed in an Italian population investigating the education and perception of the general population regarding psoriasis revealed that 7.3% of respondents have not heard of psoriasis, while 6% consider it to be a contagious condition [22].

Other studies in the field of psychodermatology found that patients with psoriasis who had lower levels of education had a greater likelihood of experiencing stigmatization and presented with higher degrees of stigmatization [23,24]. However, in the context of this particular investigation, we did not uncover any statistically significant connections between levels of education and stigmatization. The sole link that met the criterion for statistical significance was discovered for DLQI scores, which showed that patients with lower levels of education had worse quality of life on average than those with greater levels of.

Psoriasis may have a variety of consequences on a person’s mental health due to the fact that every patient reacts differently to the symptoms of their condition. Patients who are unable to deal with their illness may try suicide if they have severe psoriasis, which may be a trigger for depression [25]. Psoriasis itself may also be a motivation for patients to attempt suicide. This argues for the need for more research into the relationship between psoriasis and quality of life. This future study should give a comprehensive and holistic understanding of the condition of individuals who have psoriasis. This understanding should go beyond an evaluation of the severity of the illness and its impact on clinical status.

A recent study showed that 51.1% of patients with psoriasis feel that society does not show tolerance for their condition, 57% of patients have received uncomfortable questions regarding their condition and 59.1% report that they have experienced discrimination because of psoriasis [8]. In another paper, approximatively 66% of patients report that they are perceived as contagious and unattractive by society at large. One third of patients anticipate that their lesions would be met with negative emotional responses, such as disgust [26,27]. This is mirrored in our study as over a third of respondents report feeling pity when interacting with patients with visible psoriasis lesions. While pity is not perceived as a negative emotion in general, pity can only be felt from a position of superiority, and thus, it can be classified as a negative emotion.

Lastly, more researchers observed that individuals with psoriasis who were female had a worse quality of life and were more likely to be stigmatized than patients with the condition who were male [28]. However, other researchers found that gender did not have a major role in determining quality of life [29]. In the current research, we found that the amount of stigmatization experienced by male patients was much greater than that experienced by female patients, although the quality of life experienced by female and male patients was almost same.

### 4.2. Study Limitations

This study was performed with a significant sample from three regions of Romania, allowing for reliable statistical analysis with statistical power. One limitation of the present study lies in the group sample size and sampling bias since all three cities in which questionnaires were distributed are university cities, with their populations being relatively more educated than other locations. The difficulty of finding a homogeneous sample devoid of comorbidities and other crises that may have served as confounders could be a drawback of the research. Another weakness of this research is the cross-sectional study design, since a various of factors can influence the honesty of patients who complete paper-based and online surveys.

## 5. Conclusions

The current findings contribute to understanding the phenomenon of misinformation and stigmatization related to patients with psoriasis, enabling the development of campaigns aimed at educating the general population, increasing visibility and creating a more accepting social context for patients suffering from psoriasis. Even if respondents are relatively knowledgeable of psoriasis, a degree of stigma persists, most likely owing to unconscious emotional reactions such as repulsion and embarrassment. These patients are more likely to develop depression or depressive symptoms when their beliefs regarding psoriasis are not correctly addressed.

## Figures and Tables

**Table 1:** Comparison of background data from patients and controls.

Questions (Yes)	Patients (*n* = 218)	Controls (*n* = 374)	*p*-Value *
Age group			0.132
18–30	103 (37.1%)	166 (44.4%)	
30–50	98 (35.3%)	109 (29.1%)	
>50	77 (27.7%)	99 (26.5%)	
Gender (Men)	132 (60.6%)	204 (54.5%)	0.154
Area of residence (urban)	128 (58.7%)	210 (56.1%)	0.542
Relationship status (married)	134 (61.5%)	261 (69.8%)	0.038
Level of income (average or higher)	121 (55.5%)	227 (60.7%)	0.215
Level of education (higher education)	140 (64.2%)	259 (69.3%)	0.207
Occupation (employed)	144 (66.1%)	276 (73.8%)	0.045
Substance use behavior			
Frequent alcohol consumption	21 (9.6%)	39 (10.4%)	0.757
Frequent smoker	58 (26.6%)	74 (19.8%)	0.054
Recreational drug use	7 (3.2%)	9 (2.4%)	0.560
History of major depression or current depressed mood or anhedonia	42 (19.3%)	49 (13.7%)	0.044

* Chi-square or Fisher’s exact test.

**Table 2:** Comparison of misinformation and stigmatization as viewed by patients and controls.

Questions (Answer—Yes)	Patients (*n* = 218)	Controls (*n* = 374)	*p*-Value *
Do you know someone with psoriasis?	162 (74.3%)	157 (42.0%)	<0.001
Would you like having a friend with psoriasis?	194 (89.0%)	311 (83.2%)	0.053
Is it safe to shake hands with someone with psoriasis?	205 (94.0%)	269 (71.9%)	<0.001
Do you think psoriasis lesions are contagious?	28 (12.8%)	67 (17.9%)	0.104
Do you think that people with psoriasis have lack of hygiene?	47 (21.6%)	118 (31.6%)	0.008
Would you hire someone with psoriasis for hand working jobs?	176 (80.7%)	206 (55.1%)	<0.001
Do you believe psoriasis can be cured?	53 (24.3%)	114 (30.5%)	0.107
Are patients with psoriasis more likely to develop skin cancer?	29 (13.3%)	67 (17.9%)	0.142
Is psoriasis caused by sun exposure?	16 (7.3%)	50 (13.4%)	0.024
Do you think psoriasis is genetically transmitted?	44 (20.2%)	93 (24.9%)	0.192
Do you think psoriasis is sexually transmitted?	11 (5.0%)	64 (17.1%)	<0.001
Do you feel repulsion when you see psoriatic lesions?	20 (9.2%)	59 (15.8%)	0.001
Do you think patients with psoriasis stigmatized?	81 (37.2%)	102 (27.3%)	0.012

* Chi-square or Fisher’s exact test.

**Table 3:** Comparison of COPE-60 questionnaire results by age group.

Questions (Yes)	Likelihood *	18–30 Years (*n* = 103)	30–50 Years (*n* = 98)	>50 Years (*n* = 77)	*p*-Value *
Engagement					0.139
	Low (1–2)	54 (52.4%)	47 (48.0%)	48 (37.7%)	
	High (3–4)	49 (47.6%)	51 (52.0%)	29 (62.3%)	
Disengagement					0.040
	Low (1–2)	39 (37.9%)	30 (30.6%)	38 (49.4%)	
	High (3–4)	64 (62.1%)	68 (69.4%)	39 (50.6%)	
Emotion-focused					0.020
	Low (1–2)	36 (35.0%)	43 (43.9%)	34 (55.8%)	
	High (3–4)	67 (65.0%)	55 (56.1%)	43 (44.2%)	
Problem-focused					0.026
	Low (1–2)	43 (41.7%)	46 (46.9%)	21 (27.3%)	
	High (3–4)	60 (58.3%)	52 (53.1%)	56 (72.7%)	

* Chi-square or Fisher’s exact test; Coping Orientation to Problems Experienced Inventory (COPE-16).

**Table 4:** Comparison of physical and mental quality of life scores based on the SF-12 questionnaire stratified by age group.

Physical and Mental Health	18–30 Years (*n* = 103)	30–50 Years (*n* = 98)	>50 Years (*n* = 77)	*p*-Value *
Physical (Mean ± SD)	52.1 ± 8.1	56.7 ± 9.5	49.3 ± 8.4	<0.001
Mental (Mean ± SD)	51.2 ± 9.3	55.6 ± 10.4	57.0 ± 9.2	<0.001
Total Score (Mean ± SD)	53.3 ± 9.0	57.9 ± 8.3	55.8 ± 8.7	0.001

* Analysis of Variance (ANOVA); 12-Item Short Form Survey (SF-12).

**Table 5:** Comparison of Dermatology Life Quality Index (DLQI) results stratified by age group.

Answers (a lot & Very Much)	18–30 Years (*n* = 103)	30–50 Years (*n* = 98)	>50 Years (*n* = 77)	*p*-Value *
Item 1 (sore, itchy, painful)	22 (21.4%)	17 (17.3%)	24 (31.2%)	0.088
Item 2 (embarrassment)	37 (35.9%)	21 (21.4%)	18 (23.4%)	0.046
Item 3 (shopping/home)	9 (8.7%)	5 (5.1%)	5 (6.5%)	0.588
Item 4 (clothes)	13 (12.6%)	16 (16.3%)	9 (11.7%)	0.625
Item 5 (social activities)	24 (23.3%)	13 (13.3%)	8 (10.4%)	0.041
Item 6 (sport)	7 (6.8%)	10 (10.2%)	14 (18.2%)	0.052
Item 7 (working/studying)	16 (15.5%)	9 (9.2%)	11 (14.3%)	0.374
Item 8 (interpersonal problems)	20 (19.4%)	14 (14.3%)	14 (18.2%)	0.610
Item 9 (sexual difficulties)	12 (11.7%)	18 (18.4%)	22 (28.6%)	0.015
Item 10 (treatment difficulties)	6 (5.8%)	6 (6.1%)	10 (13.0%)	0.152

* Chi-square or Fisher’s exact test; Feelings of Stigmatization Questionnaire, Dermatology Life Quality Index (DLQI) language; The test compares the proportion of patients who answered with “a lot” or “very much” on the DLQI questions.

**Table 6:** Correlation analysis by age group.

Factors	18–30 Years (Depression)	30–50 Years (Depression)	>50 Years (Depression)
Level of education (higher education)	0.282 *	0.147	0.159
Believing that psoriasis lesions are contagious	0.103	0.055	0.127 *
Believing psoriasis can be cured	−0.312 *	−0.263 *	−0.078
Believing psoriasis is sexually transmitted	0.356 *	0.141	0.203 *
Believing psoriasis can cause skin cancer	0.418 *	0.236 *	0.311 *
Having repulsion towards psoriatic lesions	0.130 *	0.143	0.056
Feeling stigmatized	0.328 *	0.376 *	0.251 *
Disengagement coping (COPE-60)	0.146	0.273 *	0.163
Emotion-focused coping (COPE-60)	−0.198 *	0.035	0.104
Low mental health score (SF-12)	0.225 *	0.210 *	0.172
Low physical health score (SF-12)	0.137	0.143	0.356 *
Item 2 (embarrassment)	0.359 *	0.228 *	0.202 *
Item 5 (social activities)	0.314 *	0.225	0.168
Item 9 (sexual difficulties)	0.414 *	0.367 *	0.314 *

* Statistically significant Spearman’s Rank correlation coefficient rho (lower than the significance threshold 0.05).

## Data Availability

The data presented in this study are available on request from the corresponding author.
